# Patho- physiological role of BDNF in fibrin clotting

**DOI:** 10.1038/s41598-018-37117-1

**Published:** 2019-01-23

**Authors:** Patrizia Amadio, Benedetta Porro, Leonardo Sandrini, Susanna Fiorelli, Alice Bonomi, Viviana Cavalca, Marta Brambilla, Marina Camera, Fabrizio Veglia, Elena Tremoli, Silvia S. Barbieri

**Affiliations:** 1 Centro Cardiologico Monzino, IRCCS, Milan, Italy; 2 Dipartimento di Scienze Farmacologiche e Biomolecolari, Università degli Studi di Milano, Milan, Italy

## Abstract

Circulating levels of Brain Derived Neurotrophic Factor (BDNF) are lower in coronary heart disease (CHD) than in healthy subjects and are associated with coronary events and mortality. However, the mechanism(s) underling this association is not fully understood. We hypothesize that BDNF may influence fibrin fiber structure and clot stability, favoring clot lysis and thrombus resolution. We showed that recombinant BDNF (rh-BDNF) influenced with clot formation in a concentration-dependent manner in both purified fibrinogen and plasma from healthy subjects. In particular, rh-BDNF reduced the density of fibrin fibers, the maximum clot firmness (MCF) and the maximum clot turbidity, and affected the lysis of clot. In addition, both thrombin and reptilase clotting time were prolonged by rh-BDNF, despite the amount of thrombin formed was greater. Intriguingly, CHD patients had lower levels of BDNF, greater fibrin fibers density, higher MCF than control subjects, and a negative correlation between BDNF and MCF was found. Of note, rh-BDNF markedly modified fibrin clot profile restoring physiological clot morphology in CHD plasma. In conclusion, we provide evidence that low levels of BDNF correlate with the formation of bigger thrombi (*in vitro*) and that this effect is mediated, at least partially, by the alteration of fibrin fibers formation.

## Introduction

Brain-derived neurotrophic factor (BDNF), a member of neurotrophin family consisting of 118 amino acids, with a molecular weight of ∼14 kDa and a high charge (pI [isoelectric point] = ∼9–10)^1^, promotes growth, survival, and maintenance of neurons^2^. It is expressed not only in neurons, but also in other types of cells including endothelial cells^3^, cardiomyocytes^4^, vascular smooth muscle cells^5^, leukocytes^6^, platelets^7^ and megakaryocytes^8^. BDNF plays a key role not only in the brain but also in the cardiovascular system, and its alterations have been related to pathological changes in the cardiovascular system, suggesting its potential role in the pathogenesis of coronary heart disease (CHD). Indeed, BDNF promotes development of cardiac vasculature, modulates cardiac endothelium survival and proliferation, enhances capillary formation, sustains angiogenesis and maintains integrity of vascular system^9^. Interestingly, modifications of BDNF gene, including rs10767664 and rs 6265 polymorphisms, are associated with coronary artery disease and myocardial infarction^10–12^. Similarly, lower circulating BDNF levels are detected in cardiovascular disease^13,14^ and related disorders^15^, are associated with increased risk of atrial fibrillation and with future coronary events^16^, and are an independent predictor of 4-year coronary and all-cause mortality^17^. Finally, circulating BDNF is negatively associated with triglyceride, LDL-cholesterol and fibrinogen, and positively associated with HDL-cholesterol in patients with angina pectoris^18^.

However, the relationship between circulating levels of BDNF and CHD has not been fully understood as yet.

Accumulating evidence shows that both hypercoagulable state and hypo-fibrinolytic conditions are related to the increased risk of cardiovascular events and severity of CHD^19–23^, suggesting that the unbalance between clotting and lysis proteins plays an important role into CHD pathogenesis. In particular, higher plasma levels of fibrinogen as well as fibrin clot properties predict adverse clinical outcome in CHD patients and are associated with the extent of coronary atherosclerosis^24–27^. Similarly, an inadequate function of fibrinolytic system, with lower plasma levels of tissue plasminogen activator (tPA) and greater concentration of plasminogen activator inhibitor type 1 (PAI-1), has been observed in coronary artery disease patients^28^.

Interestingly, tPA not only catalyzes the conversion of plasminogen to plasmin, the key modulator of fibrin clot degradation, but also regulates the proteolytic cleavage of pro-BDNF to mature BDNF^29,30^, suggesting the fibrinolytic system as a potential bridge between BDNF and CHD. Indeed, in pathological conditions, circulating BDNF is positively associated with tPA/plasmin activity^31^ and negatively with fibrinogen^17^.

In addition, since fibrin(ogen) binds BDNF by its heparin-binding domain and since fibrin matrix retains BDNF into the clot^32^, we hypothesized that BDNF may modify fibrin fiber structure and affect clot stability favoring clot lysis and thrombus resolution.

## Methods

### Study population

Twenty one healthy men (age between 29 and 78 years) with normal sinus rhythm, no electrocardiographic alterations and history of atrial fibrillation were screened from those attending the clinic for global control of cardiovascular risk at Centro Cardiologico Monzino, IRCCS. Forty-one coronary heart disease (CHD, age between 39 and 79 years) patients, candidate for coronary artery bypass grafting (CABG), were also recruited at Centro Cardiologico Monzino. Preoperative inclusion criteria were: need for elective, isolated surgical procedure, age between 18–80 years, ejection fraction >30%, normal sinus rhythm and no history of atrial fibrillation. Individuals suffering from renal or liver disease or taking antioxidants within 30 days prior to surgery were excluded. Clinical and demographical features of controls and CHD patients are listed in Supplementary Table 1.

Blood was collected from CHD patients the day of in-hospital admittance while controls underwent sample collection at a scheduled visit. Both CHD and control subjects were not under anticoagulant drugs.

The study complies with the Declaration of Helsinki and was approved by the Institutional Review Board and Ethical Committee of Centro Cardiologico Monzino IRCCS. All participants provided written informed consent.

### Biochemical analysis

Peripheral blood sample was collected from patients and controls while fasting into a vacutainer tubes containing EDTA (ethylenediaminetetraacetic acid) disodium salt (9.3 mM; Vacutainer System, Becton Dickinson, Franklin Lakes, NJ, USA.) for detection of BDNF and fibrinogen, or into a vacutainer tubes containing Sodium Citrate (0.105 M; Vacutainer System, Becton Dickinson, Franklin Lakes, NJ, USA) for clot and thromboelastographic analysis, and then centrifuged within 30 min at 3000 g for 10 min at 4 °C. Plasma thus obtained was collected, aliquoted and immediately stored at −80 °C until analysis.

BDNF and fibrinogen were measured in plasma by kits commercially available: BDNF by Emax Immunoassay system (Promega, Madison, WI, USA), and Human FG (Fibrinogen) by ELISA assay (Whuan Fine Biotech Co., China), respectively^33^.

### Clot analysis

Fibrin clot characterization was performed (a) in a purified system, (b) in five different plasma pools obtained mixing four by four plasma from 20 healthy subjects, and (c) in 9 and 12 plasma pools from control subjects and CHD patients, respectively, obtained mixing two or three plasma, accordingly to plasma BDNF levels (±20 pg/ml) to minimize biological variability. (d) The effect of rh-BDNF on fibrin clot structure in CHD patients was performed directly in plasma sample of 12 CHD patients with BDNF < 100 pg/ml as specified in figure legends.

### Fibrin clots analysis

Fibrin clot structure: Fibrinogen-Alexa Fluor 488 conjugated (fibrinogen-AF, Invitrogen, St Louis, MO) was reconstituted in sodium bicarbonate 0.1 M pH 8.3 at a concentration of 1.5 mg/ml by layering the protein on top of the saline solution and solubilizing it with occasional gentle mixing. Then fibrinogen was aliquoted and frozen until the use. Fibrin clots were prepared in chambered coverslips using 100 µl of clotting solution with the following final concentrations: (a) In purified systems, the experiments were performed without adding of Factor XIII^34^ to focus exclusively on the reaction of BDNF on fibrin mesh. In particular, clots were prepared in HEPES buffer (HEPES 20 mM pH 7.4, NaCl 110 mM) with 1 mg/ml fibrinogen, 2.5 mM CaCl_2_ and 0.1 U/ml human thrombin (SIGMA-Aldrich, St Louis, MO) in the presence of 1 µl of PBS containing different concentrations (0–300 pg/ml) of recombinant BDNF (rh-BDNF, prepared according to manufacturer instructions in distilled water; Invitrogen, Waltham, MA, USA) or an equal volume of PBS containing BSA (1 mg/ml: control). (b) Citrated Plasma samples were diluted 1:10 in imidazole buffer^35^, then 1 µl of PBS containing scalar concentration of rh-BDNF (as indicated into the figure legend) plus or equal volume of PBS containing BSA (1 mg/ml: control) and then 10 µl of 1 mg/ml fibrinogen-AF were added. After 5 min of incubation at 37 °C, clot formation was induced by 2.5 mM CaCl_2_ and 0.1 U/ml human thrombin^36^.

The samples were allowed to polymerize for 2 hours in incubator at 37 °C, and then the images were acquired using apotome microscope (Carl Zeiss, Milano, Italy) or laser scanning confocal microscope (LSM710, Carl Zeiss, Milano, Italy) at 20X magnification. Quantization of fibers density was accomplished in blind by Image J software program according to method described previously with some modifications. Briefly, “the density of the fibers in each slide of Z-stack acquisition were measured after entering the “Image” menu, clicking on the “Adjust” box and isolating the area covered by fibers using “Threshold” tool. The threshold was automatically adjusted until the entire green area was highlighted in red. Then, measurement of the threshold area was performed as follows: we entered the set measurement dialog under the “Analyze” menu, and after checking the “Area”, “Integrity Intensity” and “Limit to Treshold”, we have clicked the “Measurement” button under the “Analyze” menu, and data have been found in the “Results” Window”^37^.

Fibrin polymerization assay: 100 µl of citrated plasma samples, diluted 1:2 in imidazole buffer, were pre incubated for 15 min at 37 °C with 1 µl of PBS containing scalar concentrations of rh-BDNF (0, 60 and 120 pg/ml) or with equal volume of PBS containing BSA (1 mg/ml: control). Plasma clotting was initiated by Thrombin (1 U/ml) and CaCl_2_ (2.5 mM). Fibrin polymerization was assessed by TECAN spectrophotometer at 350 nm with the interval of 23 sec^38^.

For Clot lysis studies, recombinant tPA 1,8 µM (Creative Enzymes, NY, USA) was added to each sample and fibrinolysis was monitored by TECAN spectrophotometer at 350 nm with the interval of 23 sec. The lysis time: time taken for turbidity to drop by 50% from maximum as a measure of lysis potential, and maximum turbidity: turbidity refers to the scattering of light as a measure of fibrin clot density^27^.

### Clotting time

For thrombin clotting time 100 µl of citrated plasma sample were incubated (for 15 min at 37 °C) with the same volume of diluent containing scalar concentrations of BDNF (0, 60 and 120 pg/ml) or BSA (1 mg/ml: control), then, 100 µl of human thrombin (1 U/ml; SIGMA-ALDRICH, St Louis, MO, USA) were added^39^.

For reptilase clotting time measurement, 180 µl of citrated plasma sample were incubated for 15 min at 37°Cwith the same volume of diluent containing scalar concentrations of BDNF (0, 60 and 120 pg/ml) or BSA (1 mg/ml: control), then, 60 µl of reptilase (2 BU/100 ml; Creatie Enzymes, NY, USA) and 60 µl CaCl_2_ (20 mM) were added^40^.

Time to reach the formation of a stable fibrin clot was recorded. All samples were measured in triplicates.

### Thrombin generation

Whole blood (WB) was drawn from healthy subjects with a 19-gauge needle without venous stasis into citrate (1/10 volume of 0.129M sodium citrate)-containing tubes (Vacutainer, Becton Dickinson) in the presence or absence of corn trypsin inhibitor (CTI, 50 µg/ml) to inhibit the intrinsic coagulation pathway.

Thrombin generation was measured using the Calibrated Automated Thrombogram (CAT) assay (Thrombinoscope BV, Maastricht, the Netherlands) on platelet-free plasma, prepared by two sequential centrifugations at 2500 × g for 15 minutes. Triplicate plasma samples (80 µl/well) were incubated for 15 minutes with 120 pg/ml BDNF or BSA 1 mg/ml (control) in PBS in the presence of 1pM Tissue Factor and 4 µM phospholipids (PPP Low reagent, Stago) in round-bottom 96-well microtiter plates (Immulon 2HB). Thrombin generation was started by the addition of a CaCl_2_/fluorogenic substrate mixture (20 µl/well FluCa reagent).

In order to correct for inner-filter effects and substrate consumption, each thrombin generation measurement was calibrated against the fluorescence curve obtained in the same plasma to which a fixed amount of thrombin-α2-macroglobulin complex was added (Thrombin Calibrator, Thrombinoscope BV). Fluorescence was read in a Fluoroskan Ascent reader (Thermo Labsystems OY, Helsinki, Finland) equipped with a 390/460 nm filter set and thrombin generation curves were calculated with Thrombinoscope software (Thrombinoscope BV). ETP (Endogenous Thrombin Potential, nM x min), Peak Height (nM Thrombin) and Velocity Index (nM/min) were used as main parameters describing thrombin generation.

### Rotational thromboelastography

The clotting potential of plasma was evaluated by Rotational Thromboelasto- graphy (ROTEM®)^41^. Briefly, plasma pools were incubated for 15 min at 37 °C with different amount rh-BDNF (0, 60 and 120 pg/ml) or equal volume of PBS containing BSA (1 mg/ml: control) and then analyzed. Three hundred µL of plasma for each condition was recalcified with star-tem^®^ solution (20 µL CaCl_2_/HEPES buffer 200 mM) and activated with Thrombin (SIGMA-ALDRICH, St Louis, MO) 1 U/ml. Recording has been started immediately and has been allowed to proceed for 60 min according to manufacturer’s instructions. Clot characterization in terms of Maximum Clot Firmness (MCF, measure of the consistence of the clot) was assessed. Before testing, an aliquot of each condition was stored at −80 °C to determine BDNF concentration.

### Statistical analysis

Statistical analyses were performed with SAS v. 9.4 software (SAS Institute). Data were compared between groups by Wilcoxon rank-sum test. Main effects of treatment and time were analyzed by repeated measures ANOVA, followed by a paired t-test with Bonferroni correction. Partial Spearman correlation was employed to assess associations between variables adjusting for group of subjects. Values of p < 0.05 were considered statistically significant. Data are expressed as mean/ ± SEM or median and interquartile range.

## Results

### BDNF affects fibrin clot network

To evaluate the potential impact of exogenous BDNF on fibrin clot structure, Alexa Fluor 488-labeled fibrinogen in presence of recombinant BDNF (rh-BDNF) was used.

**Figure 1:**
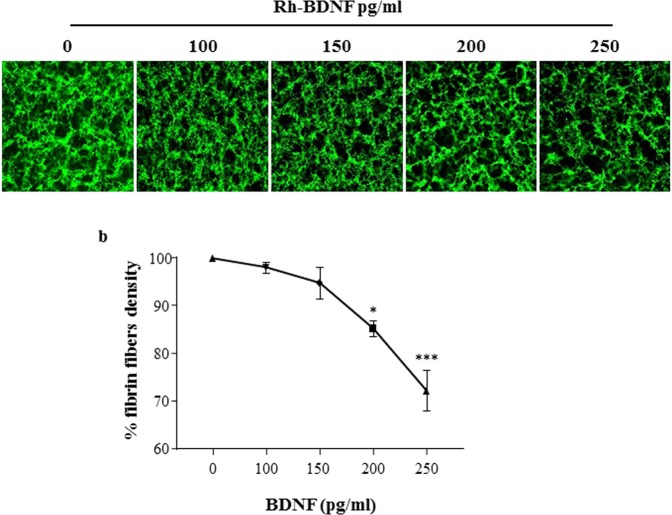
rh-BDNF reduces fibrin fibers density *in vitro*. Purified fibrin/thrombin clots were formed with recombinant BDNF (rh-BDNF: 100–250 pg/ml) or with BSA (1 mg/ml; control: point 0). (**a**) Representative image of clots using Alexa Fluor 488–labeled fibrinogen (20X magnification), and (**b**) percentage of fibrin fibers versus control. Fibrin fibers were analyzed using Image J software. All samples were performed in triplicate. Data are expressed as mean ± SEM; n = 9 *p < 0.05 ***p < 0.005.

The rh-BDNF affected the fibrin network in a concentration-dependent manner. Changes in clot structure, in terms of non-uniform, densely packed fibers and presence of large holes, are observed when 200 pg/ml rh-BDNF are added to purified fibrin(ogen)/thrombin-clot, whereas the density of fibers decreased significantly with 250 pg/ml rh-BDNF. In particular, at the highest doses used rh-BDNF reduced of about 15% and 30% fibrin fiber density compared to control (Fig. 1).

### Plasma BDNF inversely correlates with fibrin fiber density and *in vitro* clot dimension in healthy subjects’ plasma

To investigate the ability of BDNF to modify clot morphology in a physiological system, rh-BDNF was added to pools of plasma from healthy subjects. Structural analyses and polymerization of clot were assessed by visualization of fluorescent fibrin(ogen) fibers and turbidity assay, respectively, and viscoelastic properties were analysed by thromboelastography.

**Figure 2:**
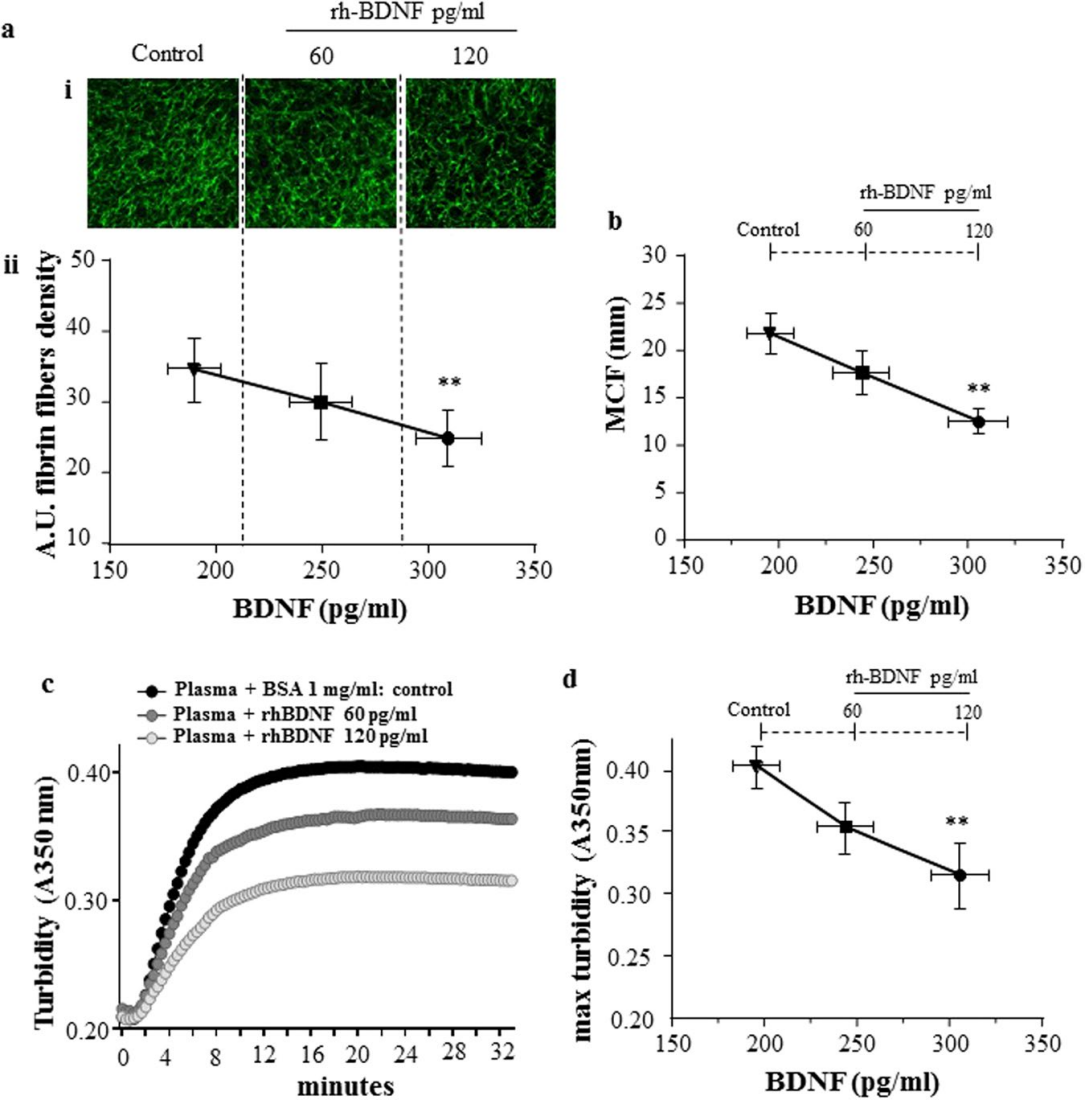
rh-BDNF influences fibrin density and polymerization, and *in vitro* clot dimension in healthy subjects’ plasma. Recombinant BDNF (rh-BDNF; 60, 120 pg/ml) or BSA (1 mg/ml: control) was added to plasma pools from healthy subjects before induction of coagulation with thrombin, then fibrin density and polymerization, and viscoelastic property of clot were analyzed. (**a**i) Visualization images (20X magnification) with Alexa Fluor 488–labeled method and (**a**ii) quantization of fibrin fibers using Image J software. (**b**) Maximum Clot firmness (MCF) assessed by thromboelastographic analyses. All samples were performed in triplicate. (**c**) Representative kinetic and (**d**) maximum turbidity detected at A350 nm at 37 °C and monitored every 23 sec by spectrophotometric method. Data are expressed as mean ± SEM; horizontal bars indicate variation of BDNF levels measured in plasma pools analyzed; n = 5 different pools. **p < 0.01.

Remarkably, a reduction of about 30% of fibrin fiber density was measured when the total amount of BDNF present in plasma (endogenous plasma levels plus rhBDNF added) reached 303.9 ± 4.93 pg/ml (Fig. 2a, and Supplementary Fig. 1a).

Similarly, rh-BDNF reduced the dimension of clot in all samples as shown by the progressive reduction in MCF (Fig. 2b, and Supplementary Fig. 1b). As expected, a positive correlation between density of fibrin fibers and MCF was found (r = 0.986, p < 0.0001, Supplementary Fig. 1c).

In addition, rh-BDNF modified the polymerization rate, defined as the slope of the turbidimetric curve, (control: 0.6198 ± 0.067, rh-BDNF 60 pg/ml: 0.547 ± 0.073 and rh-BDNF 120 pg/ml: 0.458 ± 0.053; rh-BDNF 120 pg/ml versus control p < 0.05), and the maximum optical density (Fig. 2c, and Supplementary Fig. 2a), reflecting the lateral aggregation of protofibrils and the fibre-cross-sectional area, respectively^42^.

**Figure 3:**
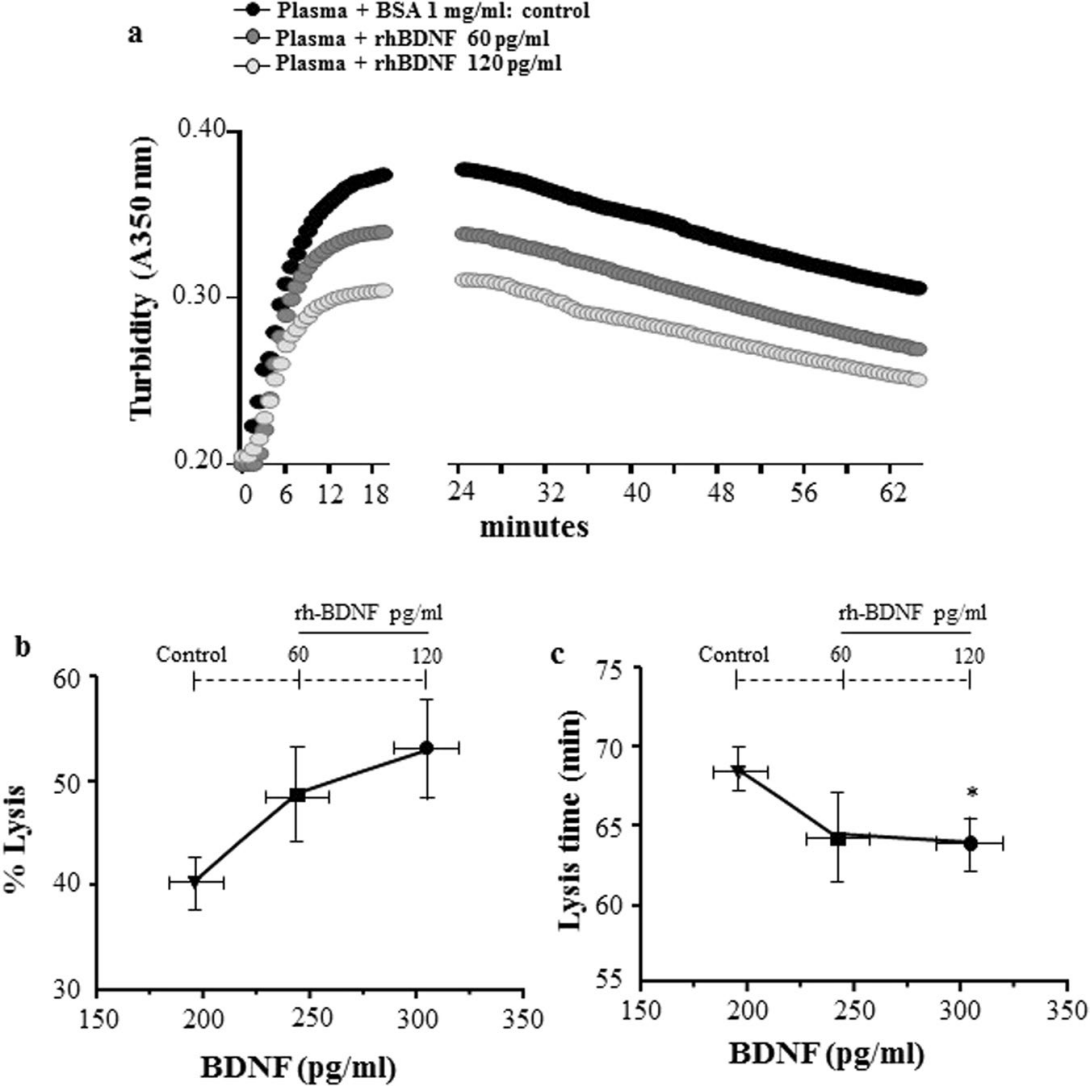
Effect of rh-BDNF on lysis of fibrin clot in healthy subjects’ plasma. Recombinant BDNF (rh-BDNF; 60, 120 pg/ml) or BSA (1 mg/ml: control) were added to five plasma pools from healthy subjects before induction of coagulation with thrombin and tPA, consequently polymerization of clot were analyzed. (**a**) Representative turbidity curves monitored by spectrophotometric method every 23 sec (A350 nm at 37 °C), (**b**) % of Lysis at 60 minutes and (**c**) Lysis time. All samples were performed in triplicate. Data are expressed as mean ± SEM; n = 5 different pools.

Rh-BDNF affected the fibrinolysis, slightly in terms of % of lysis reached at 60 minutes (control: 40.35 ± 2.48, rh-BDNF 60 pg/ml: 48.59 ± 4.71 and rh-BDNF 120 pg/ml: 53.41 ± 4.05; rh-BDNF 120 pg/ml versus control p = 0.073) and significantly the lysis time (control: 68.6 ± 1.46, rh-BDNF 60 pg/ml: 64.2 ± 3.077 and rh-BDNF 120 pg/ml: 63.5 ± 1.351; rh-BDNF 120 pg/ml versus control p = 0.043) (Fig. 3 and Supplementary Fig. 2b,c).

**Figure 4:**
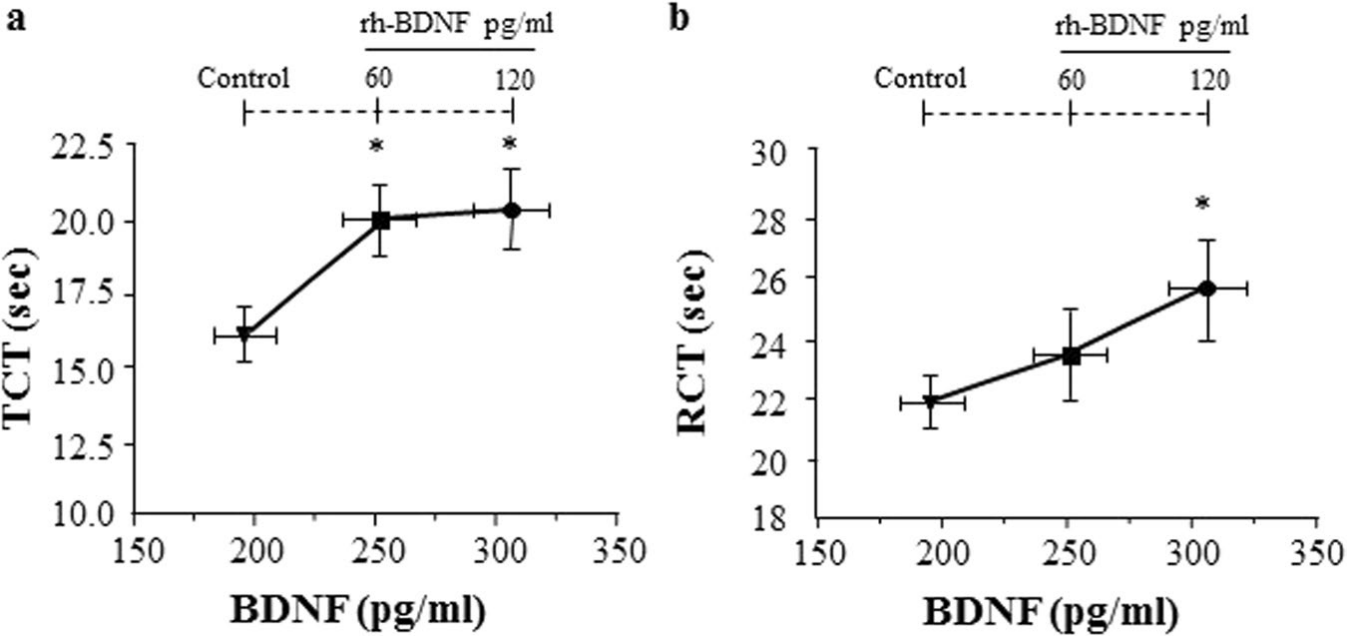
rh-BDNF influences thrombin (TCT) and e) reptilase (RCT) clotting time in healthy subjects’ plasma. Recombinant BDNF (rh-BDNF; 60, 120 pg/ml) or BSA (1 mg/ml: control) were added to plasma pools from healthy subjects, then (**a**) thrombin (TCT) and (**b**) reptilase (RCT) clotting time were measured. All samples were performed in triplicate. Data are expressed as mean ± SEM; horizontal bars indicate variation of BDNF levels measured in plasma pools analyzed; n = 5 different pools. *p < 0.05 and **p < 0.01.

Interestingly, both the concentrations of rh-BDNF prolonged thrombin clotting time (Fig. 4a and Supplementary Fig. 3a), whether only the highest concentration of rh-BDNF was able to lengthen the clotting time when experiments were performed with reptilase (Fig. 4b and Supplementary Fig. 3b).

**Figure 5:**
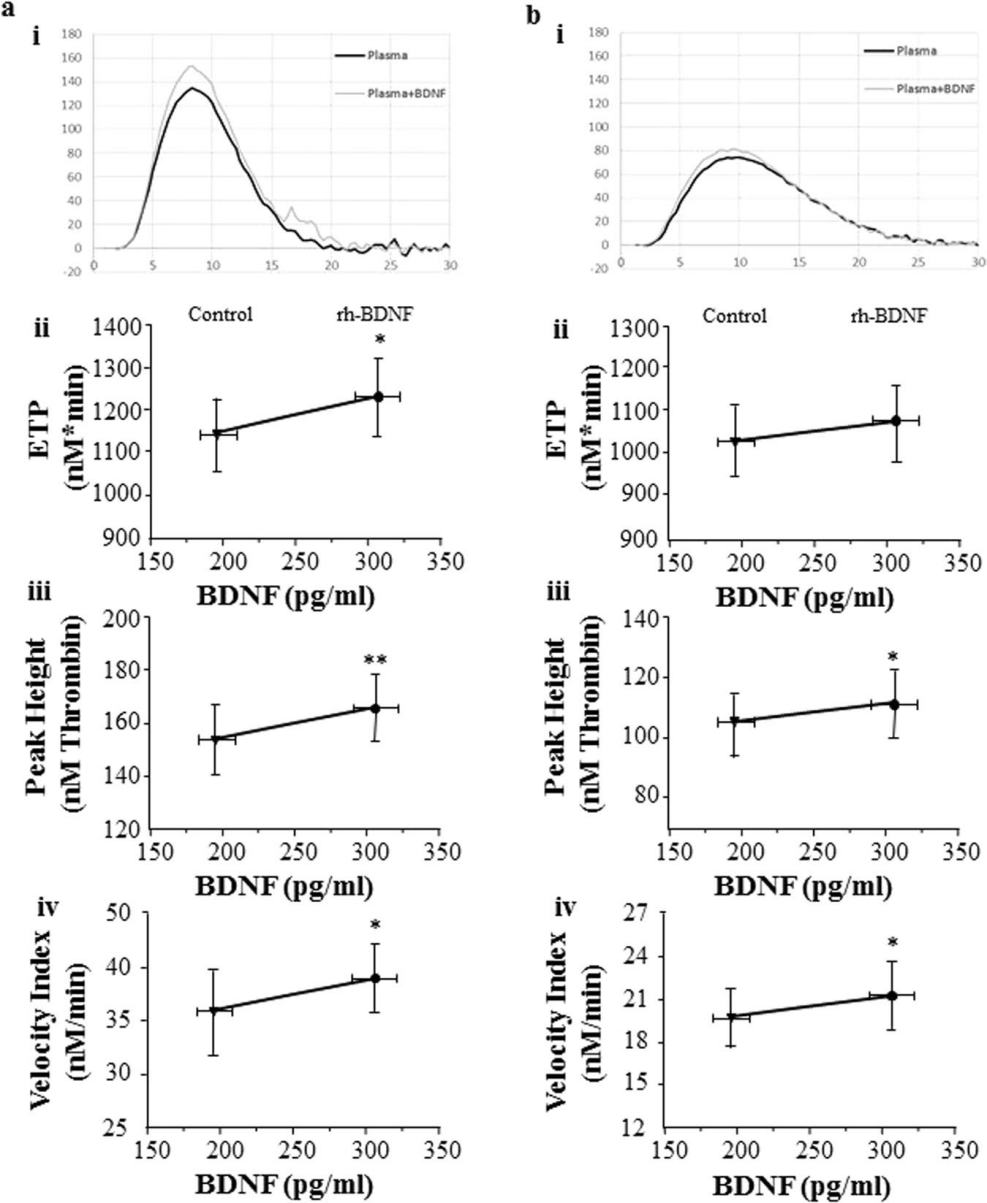
Effect of BDNF on thrombin generation. Recombinant BDNF (rh-BDNF; 120 pg/ml) or BSA (1 mg/ml: control) was added to platelet-free plasma and thrombin formation was measured by CAT assay. Thrombin generated (**a**) by the concomitant activation of both intrinsic and extrinsic coagulation pathways and (**b**) only by the extrinsic pathway. (i) Representative curves of the kinetic of thrombin formation. (ii) Endogenous thrombin potential (ETP, area under the curve), (iii) Peak Height (maximum concentration of generated thrombin) and (iv) Velocity Index (velocity of thrombin formation) were used as main parameters describing thrombin generation. Data are expressed as mean ± SEM; horizontal bars indicate variation of BDNF levels measured in plasma pools analyzed n = 5 different pools. *p < 0.05 and **p < 0.01.

Finally, the effect of BDNF on thrombin generation in human plasma from healthy donors (n = 5) was assessed by CAT assay. Data indicate that, when both the intrinsic and the extrinsic pathway are contributing to the generation of thrombin, BDNF slightly but significantly increased thrombin formation (+~8% compared to control) in all the samples tested. The ETP as well as the Peak Height and the Velocity Index were, indeed, increased in the presence of 120 pg/ml rh-BDNF compared to control samples (ETP: control: 1140 ± 195 nM x min and rh-BDNF: 1232 ± 210 nMxmin; Peak Height: control: 154 ± 29 nM and rh-BDNF: 166 ± 28 nM thromb; Vel Ind: control: 36 ± 9 nM/min and rhBDNF: 39 ± 8 nM) (Fig. 5a and Supplementary Fig. 4a).

By contrast, when the intrinsic coagulation cascade was inhibited, the TF-dependent thrombin formation was not affected by BDNF (Fig. 5b and Supplementary Fig. 4b).

### Reduced levels of BDNF in coronary heart disease patients favors the formation of bigger and more dense fiber clot

In order to assess the relationship between BDNF and fibrin clot morphology in clinical setting, firstly we measured levels of BDNF and fibrinogen in plasma of 41 coronary heart disease (CHD) patients and of 21 healthy subjects (Control).

Plasma BDNF levels were significantly lower in patients with CHD than controls (CHD: 126.83 ± 15.39 pg/ml versus controls: 208.90 ± 25.13 pg/ml; p < 0.0125; n = 41 and n = 21 patients and control, respectively), whereas fibrinogen was similar in both groups (CHD: 2.77 ± 0.63 g/l versus controls: 2.63 ± 0.51 g/l)

**Figure 6:**
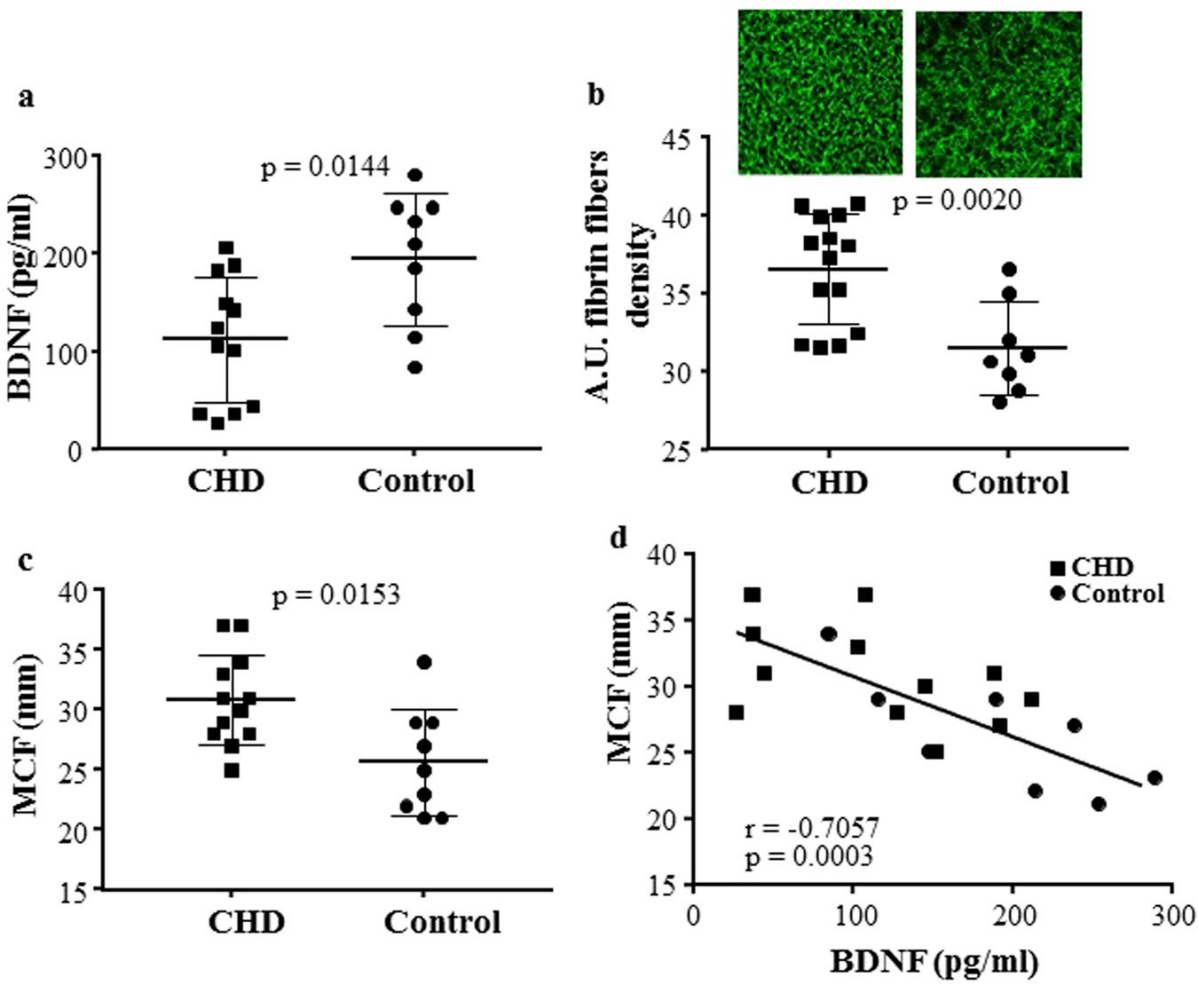
Low circulating BDNF levels are associated with higher density of fibrin fibers and greater MCF in CHD patients. Plasma pools of two or three CHD patients or healthy subjects (Control) were obtained according to similar BDNF levels (±20 pg/ml): (**a**) BDNF levels, (**b**) fibrin fibers density, and (**c**) Maximum Clot firmness (MCF) have been analysed by ELISA kit, Alexa Fluor 488–labeled method (20X magnification) and thromboelastographic analyses, respectively. Correlation between BDNF concentrations and (**d**) MCF. All samples were performed in triplicate, and representative images are shown. Data are expressed as mean ± SEM. n = 12 and 9 pools of CHD and healthy subjects, respectively.

Clot morphology analysis was carried out in pools of plasma from Control and CHD patients (see methods section). As expected, BDNF levels were lower in pools of plasma from CHD patients with respect to controls (p = 0.0144, Fig. 6a).

Interestingly, both density of fibrin fibers (p = 0.0020; Fig. 6b) and MCF (p = 0.0153; Fig. 6c) were greater in CHD than in plasma pools of controls. In addition, in the whole group (CHD and Control) a negative correlation between BDNF and MCF (r = − 0.7057, p = 0.0003) was found (Fig. 6d), and this difference remained significant after adjustment for group of subjects (r = − 0.64252, p = 0.003).

**Figure 7:**
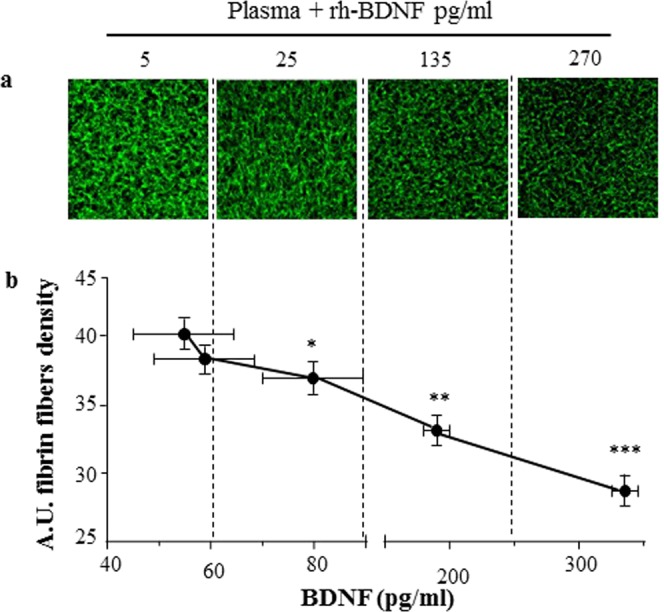
rh-BDNF reduces fibrin clot profile of CHD patients *in vitro*. Recombinant BDNF (rh-BDNF; 5, 25, 135 and 270 pg/ml) or BSA (1 mg/ml: control) was added to plasma from CHD patients before induction of coagulation with thrombin, and fibrin fibers were (**a**) visualized with Alexa Fluor 488–labeled (20X magnification) and (**b**) quantified using Image J software. All samples were performed in triplicate and representative images are shown. Data are expressed as mean ± SEM; horizontal bars indicate variation of BDNF levels measured in plasma pools analyzed. n = 12 plasma sample from CHD patients with BDNF < 100 pg/ml. *p < 0.05, **p < 0.01 and ***p < 0.005.

Of note, the addition of rh-BDNF to plasma samples of CHD patients with BDNF < 100 pg/ml markedly reduced fibrin clot profile (Fig. 4). In particular, a reduction of about 30% in density of fibrin fibers was detected when BDNF reached the concentration of about 300 pg/ml (p = 0.0017) (Fig. 7 and Supplementary Fig. 5).

## Discussion

In this study, we provide evidence that BDNF affects viscoelastic fibrin clot property and fibrin fibers density and that low levels of BDNF are associated *in vitro* with formation of bigger clot. In particular, we found that CHD patients have lower BDNF levels, higher fibrin clot strength and greater fibrin fibers complexity compared to healthy control subjects, all data consistent with previous reports^41,43^. In particular, others have showed that the range of MCF detected in control subjects and CHD patients (∼20–30 mm^44,45^ and ∼25–38 mm^45^, respectively), which confirms our data. In addition, the negative correlation between plasma BDNF levels and fibrin clot properties is suggestive of a new mechanism by which BDNF influences cardiovascular prognosis.

Several mechanisms aiming at explaining how lower BDNF levels may predict adverse cardiovascular events, have been proposed^13,14,46^. A special attention has been paid to the antioxidant, pro-survivor and pro-angiogenic effects of BDNF. Indeed, recent evidence suggests that BDNF plays a cardio-protective role by promoting the activation of enzymes involved in the detoxification of ROS^47^ and by decreasing cardiomyocytes death in hypoxic conditions^4^. In addition, it is well known that BDNF, binding to heparin-binding domain of fibrin(ogen), is retained within fibrin matrix^32^. The release of BDNF from fibrin matrix may partially explain both direct and indirect angiogenic effect of this neurothrophin. Indeed, BDNF promotes the local assembly of new vessels by induction/activation of TrkB receptor on endothelial cells^48^, and it favors the release of pro-angiogenic factors from bone marrow-derived progenitors recruited to the site of injury^49^. However, the potential involvement of BDNF in clot structure and stability has not been evaluated yet, although it is well known that BDNF binds to fragment 15–66 of fibrinogen β (Fgβ) chain^32^. This fragment is exposed after thrombin cleavage and it is involved in fibrin monomer self-association and clot formation^50^.

Interestingly, modification of Fgβ chain (e.g. tyrosine nitration and BβArg448Lys single-nucleotide polymorphism) are associated with increased fiber density and with greater resistance to fibrinolysis^51–53^. In addition, fibrin(ogen)-derived peptide β15–42 as well as fibrinogen gene deletion inhibit leukocyte infiltration, infarct size and subsequent scar formation in a mouse model of myocardial damage and reperfusion injury^54^. Overall, these findings provide compelling evidence that supports an important role of the Fgβ chain in the pathogenesis of myocardial infarction.

Thus, we can speculate that in CHD patients, *by virtue* of the lower BDNF levels, there is a great amount of free Fg β15–66 fragment that might induce stitches of cross-linking fiber with consequent generation of more dense fiber clots. In support to this hypothesis, we showed that when exogenous BDNF is added to CHD plasma, reaching physiological levels of BDNF, the density of fibrin fibers decreased as well as the amplitude of clot.

The relationship between BDNF and MCF observed in healthy subjects, as well as the ability of BDNF to affect structure, polymerization and viscoelastic properties of clot and to favor the formation of unstable clot more prone to lysis, suggests the key relevance of this neurotrophin in physiological hemostasis processes. Remarkably, the prolonged thrombin and reptilase clotting time induced by the highest concentration of rh-BDNF suggests that BDNF influences fibrin clot formation also by a mechanism independent of its binding with fibrinogen beta-chain. In particular, we can hypothesize that the high charge of BDNF might affect fibrin mesh^55,56^. Future studies will be designed in order to explain this effect.

Interestingly, the amount of thrombin generated in the presence of rh-BDNF was slightly, although significantly, increased. Thrombin plays a key role not only in the conversion of soluble fibrinogen into insoluble strands of fibrin, but catalyzes also anticoagulation related reaction, the most relevant being protein C activation. Activated protein C then inactivates FVIIIa and FVa^57,58^.

Considering the overall effect of BDNF on fibrin structure reported in the present study, it is tempting to speculate that physiological concentration of BDNF influences the thrombin-mediated anticoagulation process. This hypothesis needs to be tested in *ad hoc* studies and therefore it will be matter of future investigations.

The clinical relevance of fibrin clot properties as independent predictor of adverse clinical outcome following acute coronary syndrome has been recently highlighted by PLATO study. Indeed, Sumaya and colleagues have shown that fibrin clot properties independently predict the risk of spontaneous myocardial infarct and cardiovascular death after initial in-hospital management. In particular, enhanced resistance to lysis is associated with the increasing of established cardiovascular biomarkers (e.g. Troponin T and N-terminal pro b-type natriuretic peptide). All these findings point out to the potential need of an additional therapy in those patients with unfavorable fibrin clot structure and time of lysis^27^.

Our data would support the potential use of BDNF as biomarker of disease. It has to be considered, however, that different anti-coagulants, temperature and delay in sample centrifugation, and stability of sample storage can modify its levels^33,59,60^, thus limiting its use until standard procedures will be established.

Finally, the identification of mechanisms involved in fibrinogen modification and fibrin intrafibrillar structure are particularly attractive because the regulation of the cross-linking of the fibrin fiber as well as the preservation of endogenous thrombolytic mechanism represent crucial strategies to prevent the formation of occlusive thrombi in pathological conditions.

## Supplementary information

Supplementary Figures and Table
