# Supplementary material for: Patho- physiological role of BDNF in fibrin clotting
Source: Sci Rep. 2019 Jan 23;9:389. doi: 10.1038/s41598-018-37117-1 (PMC6344484; doi:10.1038/s41598-018-37117-1)
Supplement: Supplementary file 1 — Supplementary Figures and Table [file 41598_2018_37117_MOESM1_ESM.pdf]

Patho- physiological role of BDNF in fibrin clotting.

Patrizia Amadio[1], Benedetta Porro[1], Leonardo Sandrini[1,2], Susanna Fiorelli[1], Alice Bonomi[1], Viviana Cavalca[1], Marta Brambilla[1], Marina Camera[1,2], Fabrizio Veglia[1], Elena Tremoli[1], Silvia S. Barbieri[1*]

[1] Centro Cardiologico Monzino, IRCCS, Milan, Italy

[2] Dipartimento di Scienze Farmacologiche e Biomolecolari, Università degli Studi di Milano, Milan, Italy

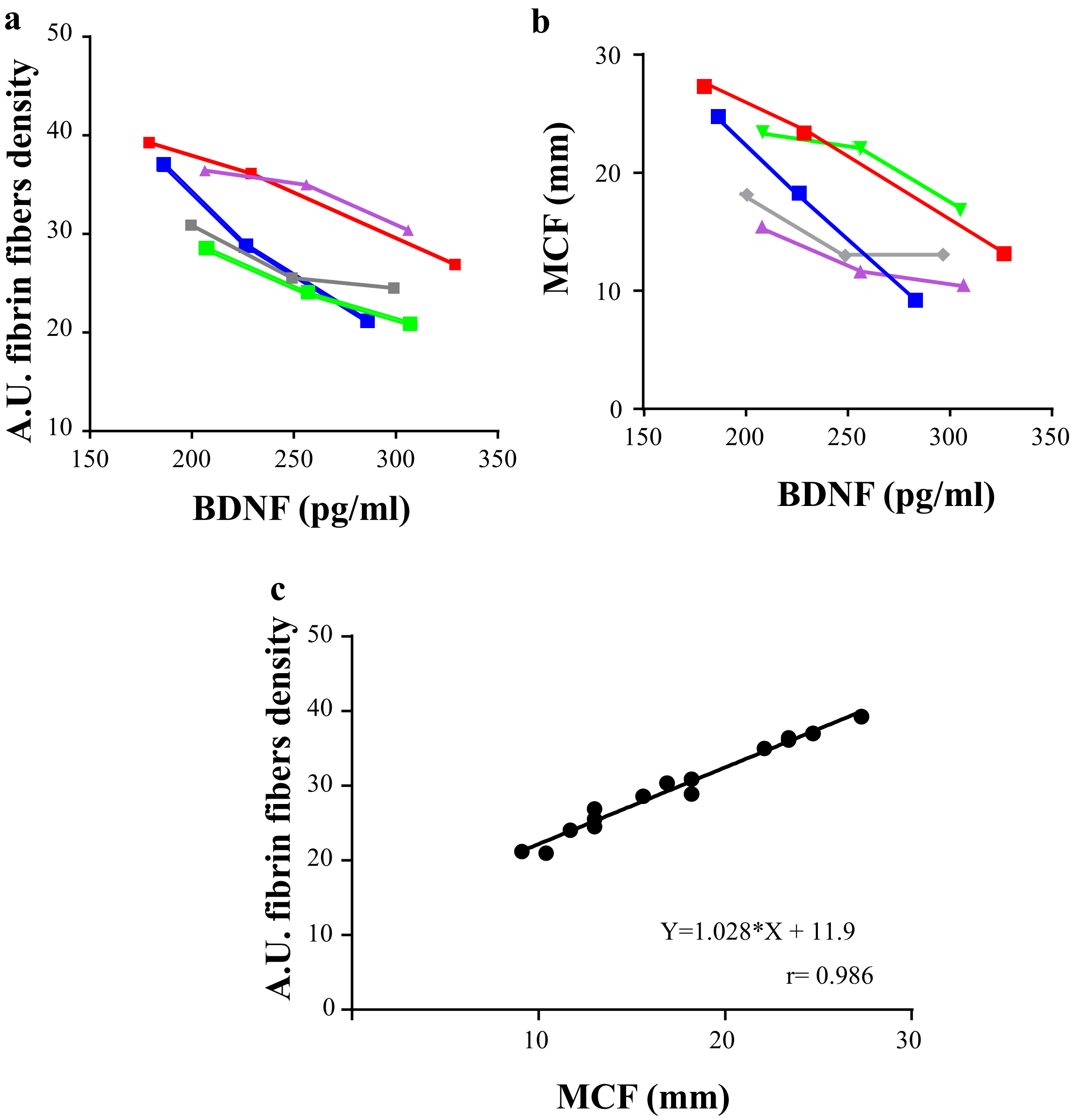

Supplementary Figure 1. rh-BDNF influences fibrin density and *in vitro* clot dimension in healthy subjects' plasma. Recombinant BDNF (rh-BDNF; 60-120 pg/ml) or BSA (1mg/ml: control) were added to five plasma pools from healthy subjects before induction of coagulation with thrombin, consequently fibrin density and viscoelastic property of clot were analyzed. **a**) Quantization of fibrin fibers with Alexa Fluor 488–labeled method, **b**) Maximum clot firmness kinetic obtained by thromboelastography. **c**) Correlation between Maximum Clot Firmness (MCF) and fibrin fibers density obtained by thromboelastographic analysis and by quantization of fluorescent fibrinogen on five pools of plasma (from healthy subjects) treated with scalar concentration of rh-BDNF. Statistical analysis was performed by Spearman rank-order correlation coefficient ($r=0.988$, $p<0.0001$; $n=15$) All samples were performed in triplicate. n=5 different pools.

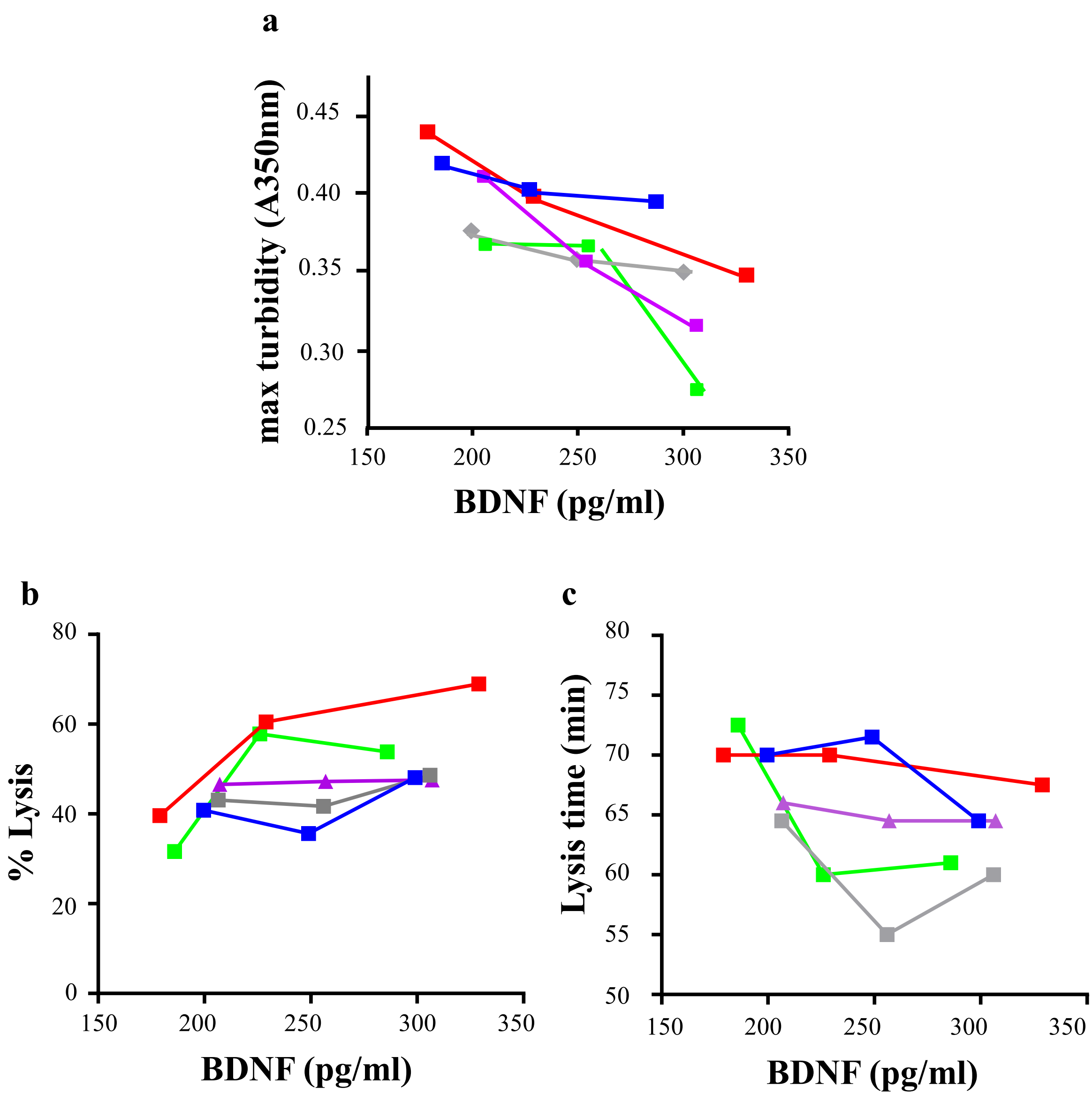

Supplementary Figure 2. Effect of rh-BDNF on polymerization and lysis of fibrin clot in healthy subjects' plasma. Recombinant BDNF (rh-BDNF; 60, 120 pg/ml) or BSA (1 mg/ml: control) were added to five plasma pools from healthy subjects before induction of coagulation with thrombin with or without tPA, consequently polymerization of clot were analyzed. **a**) Maximum turbidity, and **b**) % of Lysis at 60 minutes and **c**) Lysis time were analysed by spectrophotometric method (A350 nm at 37°C). All samples were performed in triplicate. n=5 different pools.

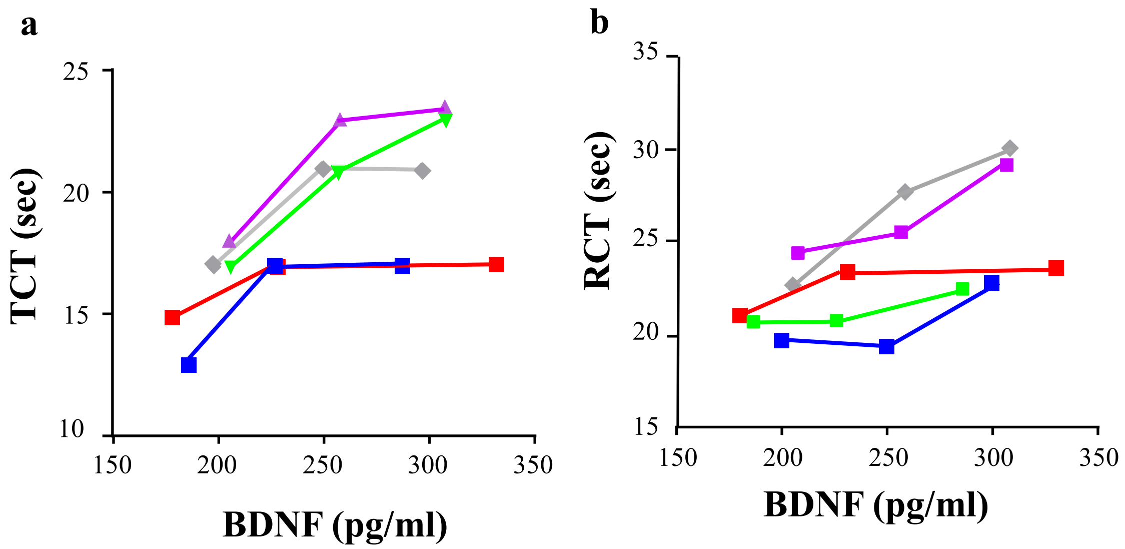

Supplementary Figure 3. rh-BDNF influences thrombin (TCT) and reptilase (RCT) clotting time in healthy subjects' plasma. Recombinant BDNF (rh-BDNF; 60, 120 pg/ml) or BSA (1 mg/ml: control) were added to plasma pools from healthy subjects and **a**) thrombin (TCT) and **b**) reptilase (RCT) clotting time were measured. All samples were performed in triplicate. n= 5 different pool.

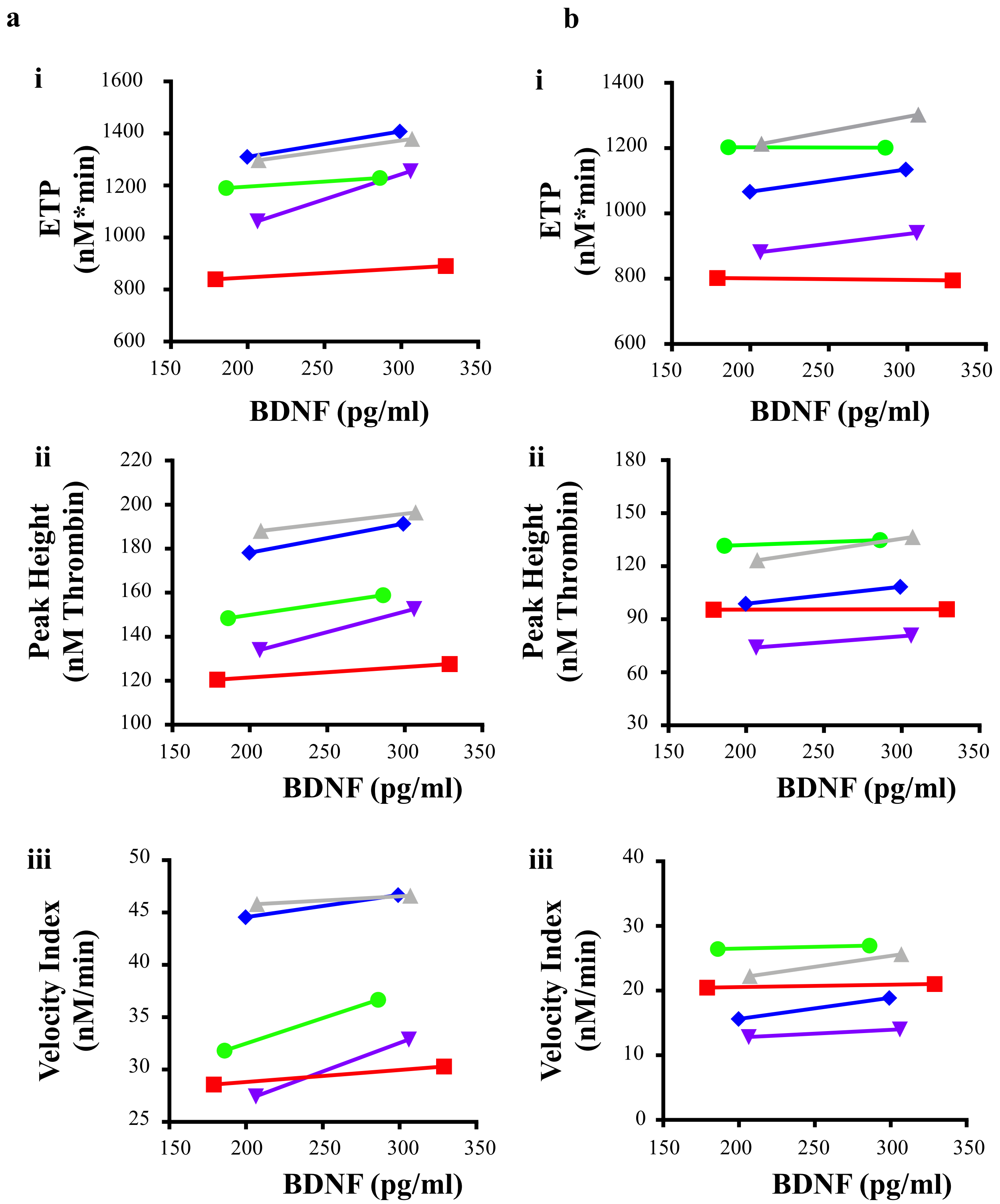

Supplementary Figure 4: Effect of BDNF on thrombin generation. Recombinant BDNF (rh-BDNF; 120 pg/ml) or BSA (1 mg/ml: control) was added to platelet-free plasma and thrombin formation was measured by CAT assay. thrombin generated **a**) by the concomitant activation of both intrinsic and extrinsic coagulation pathways and **b**) only by the extrinsic pathway. **i**) Endogenous thrombin potential (ETP, area under the curve), **ii**) Peak Height (maximum concentration of generated thrombin) and **iii**) Velocity Index (velocity of thrombin formation) were used as main parameters describing thrombin generation. n= 5.

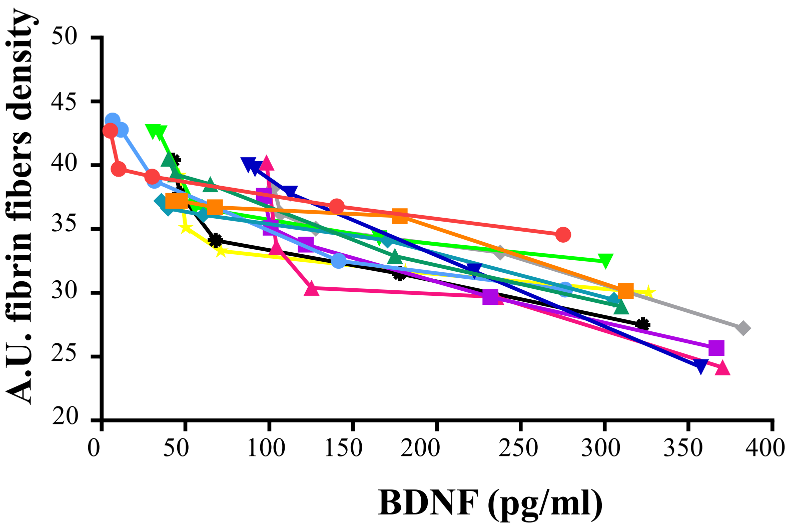

Supplementary Figure 5. rh-BDNF reduces fibrin clot profile of CHD patients *in vitro*. Recombinant BDNF (rh-BDNF 5, 25, 135 and 270 pg/ml) was added to plasma from CHD patients before induction of coagulation with thrombin, and fibrin fibers were visualized with Alexa Fluor 488–labeled. Quantification of fibrin fibers was carried out using Image J software. All samples were performed in triplicate. n= 12 plasma from CHD patients with BDNF<100 pg/ml.

Conditions	Control (n=21)	CHD (n=41)	P value
Clinical features (a)			
**Age (years)**	58.8±12.8	63.6±8.3	0.746
**BMI (kg/m^2)**	28.2±0.9	27.4±0.5	0.402
**RBC (10^6/μL)**	5.0±0.4	4.6±0.3	0.434
**WBC (10^3/μL)**	6.0±0.3	7.4±0.2	0.0002
**PLT (10^3/μL)**	216.8±11.2	203±6.3	0.250
**Total Cholesterol (mg/dL)**	223.1±9.3	175±5.6	0.0001
**HDL Cholesterol (mg/dL)**	52.2±3.9	42±1.3	0.003
**LDL Cholesterol (mg/dL)**	147.5±8.2	109±5.0	0.0001
**Triglycerides (mg/dL)**	125.9±11.0	124±8.8	0.897
**Fibrinogen (g/L)**	2.63 ± 0.51	2.77 ± 0.63	0.884
**Glycemia (mg/dL)**	108.3±5.5	123±5.7	0.104
**Smokers - n. (%)**	3/21 (14.3%)	7/41 (17.1%)	1.00
Drug treatment (b)			
**Antihypertensives - n. (%)**	3/21 (14.29%)	14/41 (34.15%)	0.136
**Hypocholesterolemics - n. (%)**	2/21 (9.52%)	29/41 (70.73%)	0.0001
**β-blockers - n. (%)**	2/21 (9.52%)	29/41 (70.7%)	0.0001
**Aspirin - n. (%)**	0/21 (0)	34/41 (82.9%)	0.0001
**Antiplatelets - n. (%)**	0/21 (0)	18/41 (43.9%)	0.0002
**Nitrates - n. (%)**	0/21 (0)	11/41 (26.8%)	0.011

Supplementary Table 1. (a) Clinical features and (b) drug treatment of control and CHD patients. BMI: body max index; RBC: red blood cells; WBC: white blood cells; PLT: platelet; HDL: high-density lipoprotein; LDL: low-density lipoprotein. Quantitative variables were expressed as mean ± SEM and categorical variables as n (%).
